# Attaining competency and proficiency in pediatric robot-assisted laparoscopic ureteric reimplantation: a learning curve configuration using cumulative sum analysis

**DOI:** 10.1007/s00345-025-05658-6

**Published:** 2025-06-14

**Authors:** Jin Kyu Kim, Nik Batra, Renee Shavnore, Konrad M. Szymanski, Rosalia Misseri, Martin Kaefer, Mark P. Cain, Joshua Roth, Pankaj Dangle, Kirstan Meldrum, Richard C. Rink, Benjamin Whittam

**Affiliations:** Department of Pediatric Urology, Riley Hospital for Children, Indiana University Health, 702 Barnhill Drive, Indianapolis, IN 46202 USA

**Keywords:** Robot-assisted laparoscopic ureteral reimplantation, RALUR, Pediatric, Learning curve, CUSUM

## Abstract

**Objectives:**

Robot-assisted laparoscopic ureteric reimplantation (RALUR) is a minimally invasive procedure for treating vesicoureteral reflux (VUR) and congenital megaureter. Despite its benefits, the adoption of RALUR involves a significant learning curve. This study aims to evaluate the learning curve of a fellowship-trained surgeon performing RALUR using cumulative sum (CUSUM) analysis.

**Methods:**

A retrospective review of RALUR cases without concurrent procedures from July 2012 to July 2024 was conducted. Patients’ clinical characteristics and surgical outcomes were assessed. The learning curve was analyzed using CUSUM for operative time (OT) and complication rates (CR), dividing the curve into three phases: learning (phase 1), competency (phase 2), and proficiency (phase 3).

**Results:**

There was a total of 65 eligible RALUR cases within specified time-period (38 bilateral). There was an overall median follow up of 16.8 months (IQR 8.2–39.4). The overall reoperation rates were 6.2% (4/65). CUSUM-OT peaked at case 23, indicating the end of the learning phase, and progression to competency (phase 2). By case 40, the surgeon achieved proficiency, with continued improvement in CUSUM-CR. Increased case complexity and trainee involvement in phase 3 did not adversely affect patient outcomes. Mention traditional OT analysis finding.

**Conclusion:**

The learning curve for RALUR can be effectively mapped using CUSUM analysis, with technical competency reached by the 24th case. Patient safety was not compromised during the learning process of trainees. Future research should include multi-institutional studies and simulation-based training to generalize findings and enhance surgical training programs.

**Supplementary Information:**

The online version contains supplementary material available at 10.1007/s00345-025-05658-6.

## Introduction

Robot-assisted laparoscopic ureteric reimplantation (RALUR) has emerged as a promising surgical technique for the treatment of vesicoureteral reflux (VUR) and other ureteral pathologies such as congenital megaureter. This minimally invasive approach aims to reduce the morbidity associated with open surgery while maintaining high success rates. Despite its advantages, the adoption of RALUR involves a significant learning curve, which can impact surgical outcomes and patient safety [1]. Nonetheless, RALUR has been shown to be as efficacious with benefits such as faster functional recovery and time to discharge for appropriately selected patients [2].

Understanding the learning curve is crucial for optimizing training programs and improving surgical proficiency for new surgeons performing this procedure in practice [3]. While there have been attempts to capture learning curves of initial case series of RALUR at several institutions, they have focused on operative times and changes in practice throughout their initial case series that improved their efficiency and outcomes [1, 4]. Hence, there has been little efforts in capturing the true numbers of cases to be considered competent or proficient in performing RALUR and does not incorporate important aspects of surgical performance, including evaluation of complication rates or evolving practice patterns.

The traditional approach to assessing the learning curve in surgical procedures involves tracking the operative time of consecutive cases performed by a surgeon. This method assumes that a reduction in operative time over successive cases reflects an improvement in the surgeon’s proficiency. The learning curve is typically represented graphically, with operative time plotted against the case number. The inflection point on this curve, where a plateau in operative time is observed, is considered indicative of the surgeon reaching a level of competence or mastery. In contrast, cumulative sum analysis (CUSUM), which has been broadly used in evaluation of a surgical learning curve, may provide a more comprehensive evaluation of a learning curve by providing holistic performance assessment focusing on both deviations of small changes in operative time and complication rates with long-term tracking of performances [5]. The CUSUM analysis evaluates performance over time by incorporating not just operative time but also complication rates. CUSUM plots are generated by calculating the cumulative sum of deviations of each operative time or complication from a predetermined target value often defined as mean operative time or acceptable/unacceptable complication rates reported in the literature. CUSUM incorporates both efficiency and safety, offering a comprehensive assessment of the learning process. Unlike the traditional operative time approach, the inflection points on CUSUM charts indicate shifts in performance, either improvements or deteriorations, that can prompt further training or adjustments in technique beyond the initial learning curve. Herein, this study aims to provide this insight by constructing and evaluating the learning curve of a fellowship-trained surgeon performing RALUR using CUSUM.

## Methods

Following institutional research ethics board approval (IRB#1605024102), a retrospective review of RALUR cases from July 2012 to July 2024 was conducted. All patients who underwent RALUR by the index surgeon (BW) since the start of their practice post-fellowship were included. Only primary procedures without concomitant procedures were considered, and redo procedures were not included in the learning curve analysis. Patients without sufficient follow up (defined as > 1 month) were excluded from the analysis. Clinical characteristics assessed included sex, age at surgery, laterality, duplexity, VUR grade, prior endoscopic bulking agent (Deflux^®^) injection, pre-existing comorbidity, body mass index (BMI) percentile, bowel and bladder dysfunction (BBD), recurrent urinary tract infections (UTI), and continuous antibiotic prophylaxis status. Indwelling stents are not placed for our RALUR technique.

All patients with VUR are counseled based on their management options, including observation with or without antibiotic prophylaxis, Deflux^®^ injection, open ureteral reimplantation, and minimally-invasive (robot-assisted laparoscopic) ureteral reimplantation. Those with persistent high-grade reflux or recurrent UTIs are counseled on intervention. Deflux^®^ is generally recommended to patients who desire minimally invasive endoscopic management and with low grade reflux for best procedural success. The best candidates for RALUR are those who are older/post-pubertal or with high BMIs; these patients are counseled to consider RALUR as it may facilitate easier dissection with less post operative pain.

**Fig. 1:**
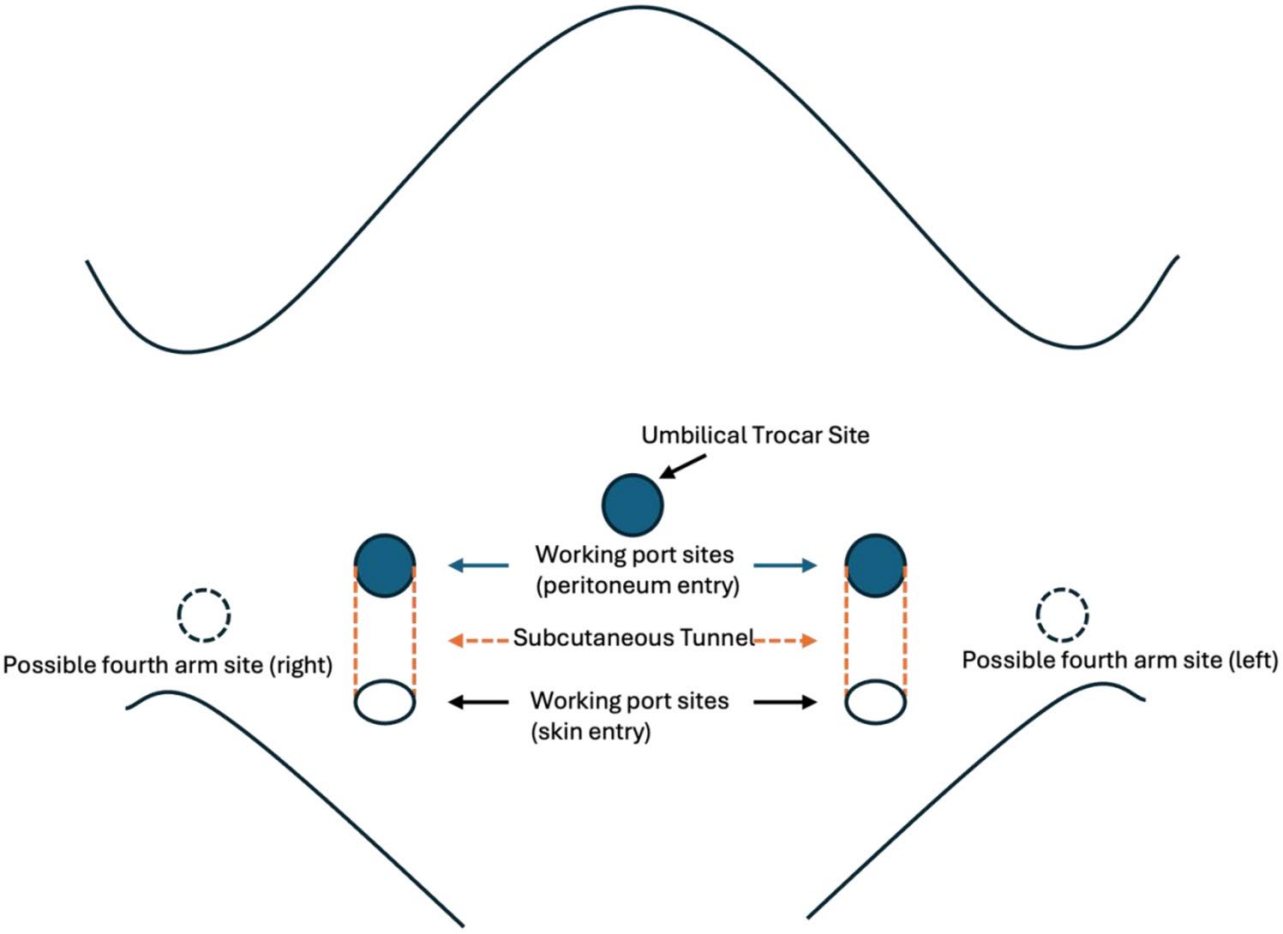
The index surgeon’s robot-assisted laparoscopic ureteral reimplantation trocar port placements

An umbilical incision is made, and pneumoperitoneum is established using a Veress needle. A 5 mm Optiview^®^ trocar is used to enter the abdominal cavity under direct visualization. Three 8 mm robotic trocars are then placed (Fig. 1), with two inserted under vision via skin incisions medial to the anterior superior iliac spine. These are tunneled superiorly through the subcutaneous tissue to enter the peritoneum above the skin incision site, enabling adherence to hidden incision endoscopic surgery principles while optimizing pelvic access. A fourth robotic arm is occasionally placed in the lower quadrant, inferolateral to the working port on the ipsilateral side of the ureteral reimplantation.

After trocar placement, the 5 mm Optiview^®^ is exchanged for a robotic camera port. The patient is positioned in Trendelenburg, and the robot is docked. The external ring is identified and traced medially to visualize the ureter at the peritoneal reflection over the bladder. A transverse peritoneal incision is made, and the ureter is carefully dissected free, avoiding reproductive organs and their vasculature. The ureter is mobilized to reach the internal ring and confirm adequate length.

The bladder is filled, and a 5 cm detrusor tunnel is created superior to the ureter. The detrusor muscle is incised to the mucosa, and flaps are fashioned without violating the mucosa. The ureter is positioned within the tunnel, and a 3–0 PDS suture is placed distally to anchor it by incorporating the adventitia. The detrusor is then closed proximally in an interrupted fashion with 3–0 PDS sutures to complete the tunnel. Tunnel patency is confirmed by passing a 5-French feeding tube between the ureter and detrusor closure.

Following the surgery, patients generally stay one night in hospital with a foley catheter. The foley catheter is removed on the morning of post-operative day 1 and monitored to ensure low post-void residuals. Patients are seen back in clinic in 1 month with a repeat renal and bladder ultrasound for reassessment. Patients are then seen at 6 months post-operatively with an ultrasound and then at 1-year post-operatively. Following this, patients are seen annually unless patient/family preference to be seen earlier or clinical concerns. Voiding cystourethrograms (VCUG) are generally not performed, unless patient/family preference to confirm resolution or recurrent febrile UTIs suggestive of clinically significant unresolved VUR.

Outcome variables included OT, worsening hydronephrosis, return to the emergency department within 30 days, readmission, repeat procedures (Clavien-Dindo classification ≥ 3 complications), and postoperative UTIs during follow-up. For the purposes of building a CUSUM analysis, the OT for bilateral cases was normalized to the unilateral cases using ratio of average bilateral OT and unilateral OT (bilateral case OT/[average bilateral OT/average unilateral OT]).

OT were used to measure the learning curve and Clavien-Dindo classification ≥ 3 complications to determine the complication rate (CR). A CUSUM analysis was performed to generate a learning curve plot, evaluating the evolution of practice in terms of OT and CR. For OT, CUSUM-OT was calculated by setting the first patient’s CUSUM-OT as the difference between their OT and the mean OT of all patients. This method was repeated for each patient until the CUSUM-OT of the last patient reached zero. In other words, the analysis is cumulative over the entire series: it is informed by every single case, regardless of its place in the sequence.

The learning curve for surgical outcomes was determined by cumulative charting of the repeat procedure rate (CUSUM-CR) across the RALUR series. Parameters for the learning curve assessment were set with α (type 1 error) at 0.05 and β (type 2 error) at 0.2. The acceptable failure rate was set at p1 = 0.05, aligning with a 95% average success rate for open ureteric reimplantation for high grade reflux, while the unacceptable failure rate was set at p1 = 0.2, based on the 80% success rate of endoscopic bulking agent injection [6, 7]. The equations used were P = ln(p1/p0), Q = ln((1 − p0)/(1 − p1)), S = Q/(P + Q), with the series unacceptable complication rate line (h1) calculated as a/(P + Q) and the acceptable complication rate line (h0) as − b/(P + Q), where a = ln((1 − β)/α) and b = ln((1 − α)/β; Appendix A) [3, 5].

In the context of CUSUM, these equations help define the control limits (h0 and h1) on the CUSUM chart. If the CUSUM statistic exceeds the h1, it indicates that the complication rate has crossed into an unacceptable range. This suggests that the surgeon’s performance may need intervention or further investigation. The h0 line serves as a lower threshold. If the CUSUM statistic stays above h0 and below h1, the surgeon’s performance is within the acceptable range. By plotting the CUSUM against these thresholds, surgeons can monitor their complication rates in real-time. This allows for early detection of trends that might suggest deteriorating performance (crossing h1) or continued acceptable performance (remaining between h0 and h1). The goal is to provide an objective and sensitive tool for maintaining high standards in surgical practice, with immediate feedback that can guide ongoing improvement efforts [3].

For both CUSUM-OT and CUSUM-CR, a decline in the graph indicates getting further from the target value (mean operative time and h0/h1) and suggests the surgeon is becoming more efficient or safer with their surgical techniques. In contrast, if there is a rising trend, it indicates getting closer to the target values and increasing operative time or complication rates in the dataset [8].

The learning curve was divided into three distinct phases [9]. Phase 1 (Learning Phase) extended from the beginning of the evaluation to the point where the CUSU M-OT plot peaked and the CUSUM-CR plot fell below the unacceptable complication rate line (h1) with both α and β values at 0.1. Phase 2 (Competency Phase) was identified when the CUSUM-CR and CUSUM-OT remained stable and plateaued. Phase 3 (Proficiency Phase) began when the CUSUM-CR continued to decline. Following prior literature, an additional aspect of the Proficiency Phase, known as the Case-Mix Phase, was included [10]. In this phase, surgeons who had already achieved competency and proficiency took on more complex cases and involved trainees more extensively. Analyses were conducted to assess differences in outcomes and case characteristics across each phase of the learning curve.

## Results

There was a total of 65 eligible RALUR cases within specified time-period (38 bilateral, 27 unilateral). There were three duplex cases (case 9, 10, 25) where common sheath extravesical reimplant was performed. There was an overall mean follow up of 16.8 months (IQR 8.2–39.4 months). The rate of post-operative UTI was 17% (11/65). No other Clavien-Dindo II complications were noted. The overall reoperation (Clavien-Dindo classification ≥ 3) rate was 6.2% (4/65). All four interventions were stent insertions for obstruction (one case also required drain insertion for fornyx rupture). The median operative time was 157 min (IQR 140–185 min; Table 1). In our cohort, there were no episodes of urinary retention following bilateral ureteral reimplantations.

**Table 1:** Baseline characteristics for patients undergoing any robot-assisted laparoscopic reimplantation (n = 65)

	Median, N	IQR, %
FU months	16.8	8.2–39.4
Female sex	9	13.8%
Age at procedure	8.9	6.7–13.0
Laterality		
Bilateral	27	41.5%
Left	23	35.4%
Right	15	23.1%
VUR grade		
1	2	3.1%
2	8	12.3%
3	28	43.1%
4	23	35.4%
5	4	6.2%
Prior deflux	3	4.6%
Comorbidity	22	33.8%
BMI %ile	75	44.7–96.1
Bowel/bladder dysfunction	30	46.2%
Recurrent UTI	55	84.6%
Continuous antibiotic prophylaxis (CAP)	50	84.7%
Duplex	7	10.8%
Normalized OR time (min)	157	139.7–185
Worsening hydronephrosis		
Improvement on observation	7	10.8%
No improvement on observation	2	3.1%
Return to ED in 30 days	3	4.6%
Readmission	1	1.5%
Repeat procedure	4	6.2%
Post-operative UTI	11	16.9%
Febrile	6	9.2%

**Fig. 2:**
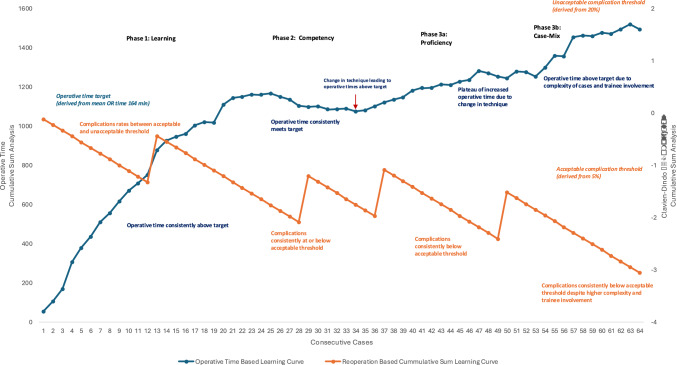
Learning curves for operative time (OT) and complication rates (CR) using cumulative sum analysis. Red arrow indicates case 34, where change in surgical technique for intradetrusor tunnel creation occurred (change from continuous suturing to interrupted suturing). Y-axes for OT indicates Represents the cumulative sum of the differences between the actual operative time and the expected/mean operative time (declining graph indicates improving operative time compared to the mean operative time) and CR indicates cumulative sum of deviations from the expected complication rate, respectively (declining graph indicates decreasing likelihood of complication compared to acceptable and unacceptable complication rates)

The fellowship-trained surgeon outcome was always below unacceptable rates of complication (h1) throughout the learning curve (Fig. 2; Supplementary Fig. 1). CUSUM-OT continued to uptrend until case 23 (phase 1, Learning Phase), where it plateaued and the surgeon achieves competency where CUSUM-CR also remains stable near acceptable complication rate, h0 (Phase 2, Competency Phase; Supplementary Fig. 2). Following case 34, the surgeon achieves proficiency as seen by continuing decreasing trend of CUSUM-CR (phase 3a, Proficiency Phase; Supplementary Fig. 3). At case 53, the surgeon’s CUSUM-OT appears to increase despite ongoing decrease in CUSUM-CR, entering the Case-Mix Phase (phase 3b; Supplementary Fig. 4).

A change in technique was implemented by the index surgeon beginning with case 34, transitioning from a running suture (placed proximal to distal) to interrupted sutures (placed distal to proximal) for submucosal tunnel creation. This modification enabled more precise control of tunnel length, as running sutures limited adjustability. Interrupted sutures also facilitated earlier trainee involvement, allowing for easier revision of suture placement. Additionally, in older children with higher voiding pressures, interrupted sutures were thought to improve tunnel integrity during healing. This technique change contributed to the increased OT observed during the learning curve. As proficiency with the new method improved, the surgeon progressed into phase 3b of the learning curve.

There was an overall similar baseline characteristics across the phases, with exception of older age in children in latter phases (p < 0.001). While not statistically significant, the cases had higher rates of cases with comorbidities in phase 3a (42.1%) and 3b (46.2%) compared to phases 1 (21.7%) and 2 (30.0%, p = 0.412; Table 2).

**Table 2:** Comparison of clinical characteristics and outcomes based on learning curve phases

	Phase 1: Learning (case 0–23)		Phase 2: Competency (24–34)		Phase 3a: Proficiency (35–52)		Phase 3b: Case-Mix (53 +)	
	Median, N	IQR, %	Median, N	IQR, %	Median, N	IQR, %	Median, N	IQR, %
Follow-up (months)	10.3	4.5–51.9	25.5	16.1–63.3	18.6	12.7–37.2	10.6	5.4–19.3
Sex (female)	21	91.3%	6	60.0%	17	89.5%	12	92.3%
Age at procedure (years)*	7.5	5.7–11.2	8.1	6.5–8.6	10.0	7.3–14.9	13.2	10.5–15.5
Laterality of reflux								
Bilateral	11	47.8%	6	60.0%	7	36.8%	3	23.1%
Left	8	34.8%	2	20.0%	6	31.6%	7	53.8%
Right	4	17.4%	2	20.0%	6	31.6%	3	23.1%
Reflux grade								
1	1	4.3%	0	0.0%	1	5.3%	0	0.0%
2	4	17.4%	1	10.0%	3	15.8%	0	0.0%
3	11	47.8%	2	20.0%	8	42.1%	7	53.8%
4	5	21.7%	7	70.0%	6	31.6%	5	38.5%
5	2	8.7%	0	0.0%	1	5.3%	1	7.7%
Prior bulking agent	1	4.3%	0	0.0%	0	0.0%	2	15.4%
Comorbidity	5	21.7%	3	30.0%	8	42.1%	6	46.2%
BMI percentile	67.0	32.0–94.0	75.0	56.0–99.0	80.8	39.09–98.5	69.75	44.7–98.8
BBD	13	56.5%	3	30.0%	9	47.4%	5	38.5%
rUTI	20	87.0%	7	70.0%	18	94.7%	10	76.9%
CAP	19	90.5%	8	80.0%	15	93.8%	8	66.7%
Duplex	3	13.0%	2	20.0%	2	10.5%	0	0.0%
OR time normalized*	190.0	159.5–214.0	136.5	127.0–143.7	150.0	137.0–158.7	149.2	137.0–167.0
Worsening hydronephrosis								
Improvement on observation	5	21.7%	1	10.0%	0	0.0%	1	7.7%
No improvement on observation	1	4.3%	0	0.0%	1	5.3%	0	0.0%
Return to ED in 30 days	1	4.3%	1	10.0%	0	0.0%	1	7.7%
Readmission	1	4.3%	0	0.0%	0	0.0%	0	0.0%
Repeat procedure	1	4.3%	1	10.0%	2	10.5%	0	0.0%
UTI post-operatively	3	13.0%	1	11.1%	6	31.6%	2	15.4%
Febrile	2	8.7%	1	11.1%	2	10.5%	1	7.7%

*Indicates significance on Kruskal–Wallis test p < 0.05

**Fig. 3:**
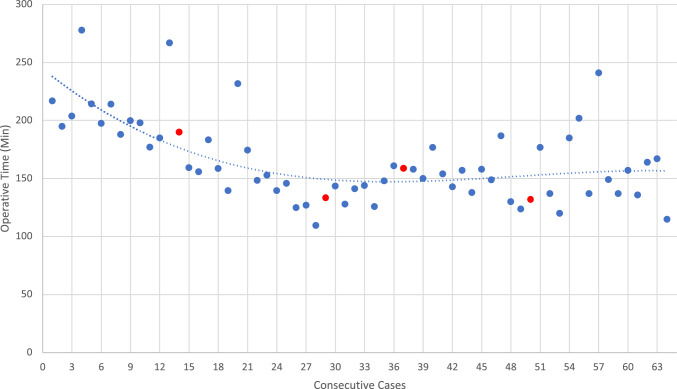
Traditional operative time-based learning curve with polynomial trend line (dotted line). Red dots indicate cases with complications

When comparing the CUSUM based learning curve to that of a traditional OT-based learning curve (Fig. 3), the traditional learning curve shows a plateau of OT at around 30 cases. While this may represent ‘competency’ based on OT alone, it fails to capture the relation of this curve to complication rates and the subtleties behind practice pattern changes with smaller fluctuations in the trendline.

## Discussion

This study provides a comprehensive analysis of the learning curve for RALUR performed by a fellowship-trained surgeon over a 12-year period. The results highlight the progression through distinct phases of the learning curve, characterized by initial increases in operative times, followed by stabilization and eventual proficiency. The learning curve analysis revealed three distinct phases: the Learning Phase (cases 1–23), the Competency Phase (cases 24–33), and the Proficiency phase (cases 34 +).

This study is the first to evaluate the learning curve of RALUR using the CUSUM method, limiting direct comparisons with existing literature. However, Esposito et al. reported continued decreases in operative and docking times up to 20 cases, aligning with our learning phase extending to case 23 [4]. In contrast, our traditional operative time (OT)-based analysis showed improvement until approximately case 30. The shorter OT in Esposito’s cohort may reflect institutional differences in technique, practice patterns, and case complexity, as no standardized method for comparing OT across centers currently exists [11].

Although literature on RALUR learning curves is sparse, some laparoscopic series suggest stabilization of OT after 6–7 cases, such as in a study of 30 laparoscopic Lich-Gregoir reimplantations [12]. However, this is not directly comparable to our robotic technique. As a single-surgeon series, our findings may not reflect broader variability due to differences in baseline skills and prior robotic experience. This limitation is common in learning curve studies. By contrast, learning curves for procedures like robot-assisted pyeloplasty are better characterized. Despite variability, multiple institutional reports suggest competency is typically achieved around 30 cases, supporting the consistency of our findings [13–16].

The CUSUM method offers advantages over traditional OT-based analyses by incorporating complication rates and enabling a dynamic assessment of surgeon performance. While OT curves may plateau and obscure ongoing changes, CUSUM reveals shifts in surgical technique, practice patterns, efficiency, and trainee involvement over time. For example, the transition from running to interrupted sutures is clearly reflected in the CUSUM plot as an increase in OT, whereas the traditional OT curve shows a plateau. This method also contextualizes performance against accepted complication thresholds; in our study, the fellowship-trained surgeon remained within acceptable limits throughout. Importantly, although the technique change resulted in increased OT, it did not negatively impact outcomes—Clavien-Dindo ≥ 3 complications, readmissions, and postoperative UTIs remained stable across all phases. This suggests surgeons in proficiency phase of their learning curve may adjust their techniques without negative consequences to patient outcomes.

Trainee participation may influence learning curves. In our series, learners were pediatric urology fellows with variable robotic experience. Unlike conventional “forward” teaching, the index surgeon employed a “backward” teaching approach [16]. Fellows began with final steps such as detrusor tunnel closure and gradually progressed to earlier steps like flap creation and ureteral dissection. This method, combined with differing trainee skillsets, likely contributes to institutional variability in the case-mix phase of the learning curve.

However, the overall complication rate in this study was comparable to previously reported rates for both robotic and open ureteric reimplantation, suggesting that the learning curve for RALUR does not compromise patient safety when performed by a fellowship-trained surgeon, despite trainee involvement in case-mix phase [6]. Interestingly, in case-mix phase, the introduction of more complex cases and increased involvement of trainees did not adversely affect outcomes and CUSUM-CR continued to downtrend, highlighting the surgeon’s ability to manage complexity and provide effective mentorship. Similar trends were seen in prior investigations with case-mix phases where despite higher trainee involvement, there was ongoing decrease in CUSUM-CR despite inevitable increase in the operative time [10]. This suggests that in a well-designed fellowship program, appropriate mentorship will continue to keep complication rates low in patients while still contributing to the training and development of the next generation of urologic surgeons.

There are several limitations to this study. The median follow-up duration was approximately 16 months, and long-term post-operative complications may not be captured in this series. Nevertheless, as the first study to utilize the CUSUM method to analyze the learning curve of RALUR in children, it offers valuable insights and benchmarks for future research and new surgeons. Since the data reflect the experience of a fellowship-trained pediatric urologist, they may not capture the learning process during residency and fellowship. To enhance generalizability, future prospective studies should include cases from early training, varying case difficulties, and prior surgical experience.

Future research should focus on comparing learning curves across different institutions and among surgeons with varying levels of prior experience. Additionally, there may be positive impacts of simulation-based training and other educational interventions on the learning curve for RALUR. We aim to collaborate with other institutions with tracked trainee/surgeon operative times to assess how this may affect the surgeon learning curve. By continuing to explore these aspects, the surgical community can develop more effective training programs and ultimately improve patient outcomes in robotic urologic surgery.

## Conclusion

Surgeons performing RALUR in children will continue to develop their skills through their early cases, achieving technical competency around the 24 th case. Ongoing perioperative optimizations can further enhance patient outcomes. For surgeons who are proficient in RALUR, trainee mentorship should be encouraged as it does not increase complication rates.

## Supplementary Information

Below is the link to the electronic supplementary material.

Supplementary file1 (PDF 82 KB)

Supplementary file2 (PDF 80 KB)

Supplementary file3 (PDF 82 KB)

Supplementary file4 (PDF 80 KB)

## Data Availability

No datasets were generated or analysed during the current study.

## Appendix A.

**Table:**

Variable/Function	Value	CUSUM equation for plot
α	0.05	95% confidence
ß	0.1	90% power
p0	0.05	5% of total cases
p1	0.2	20% of total cases
a	2.890372	ln((1 – ß)/α)
b	2.251292	ln((1 –α)/ß)
P	1.386294	ln(p1/p0)
Q	0.17185	ln((1−p0)/(1−p1))
h0	−1.44485	−b/(P+Q)
h1	1.855009	a/(P+Q)
S	0.110292	Q/P + !

^*^ln = natural logarithm
