# Supplementary material for: Attaining competency and proficiency in pediatric robot-assisted laparoscopic ureteric reimplantation: a learning curve configuration using cumulative sum analysis
Source: World J Urol. 2025 Jun 14;43(1):372. doi: 10.1007/s00345-025-05658-6 (PMC12167274; doi:10.1007/s00345-025-05658-6)
Supplement: Supplementary file 1 — Supplementary file1 (PDF 82 KB) [file 345_2025_5658_MOESM1_ESM.pdf]

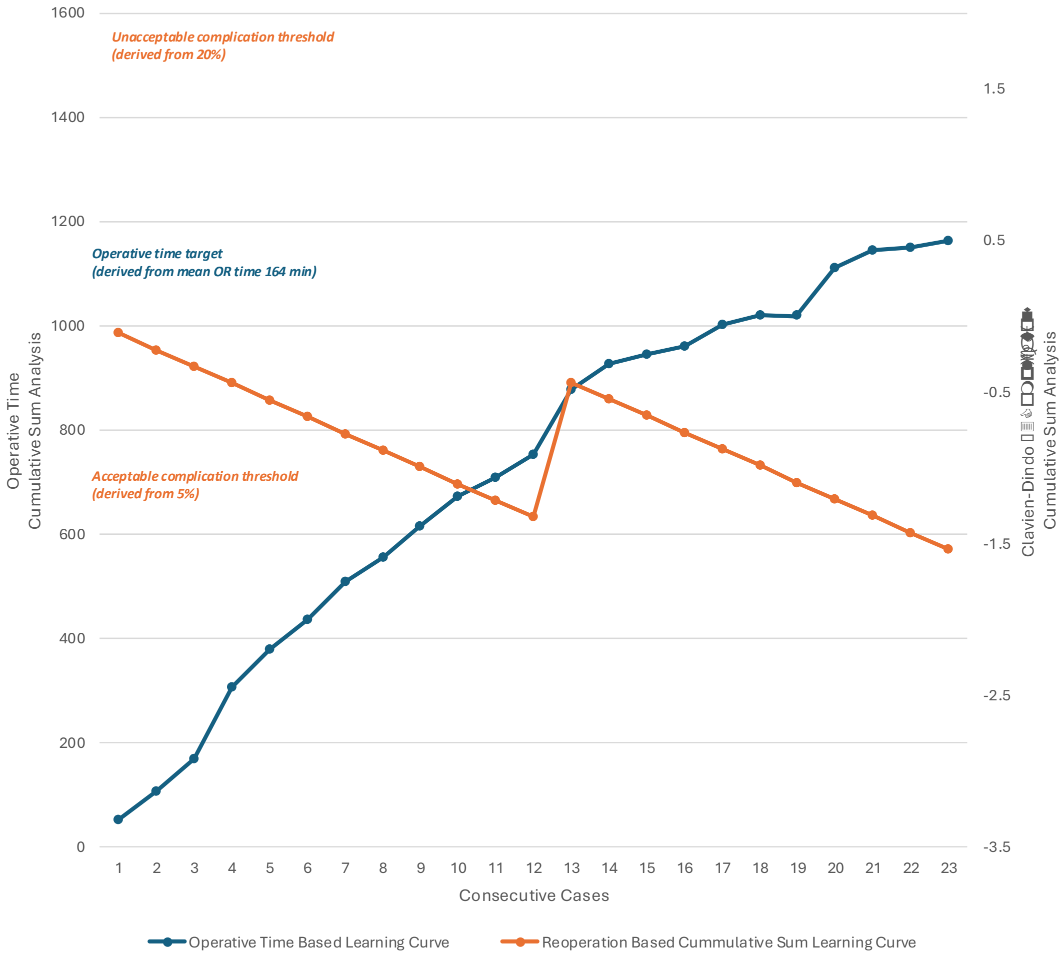

Supplementary Figure 1. Close-up detailed view of learning phase of the learning curve; complication rates are between acceptable and unacceptable threshold and operative time consistently above target (upwards trend)
