# Supplementary material for: Attaining competency and proficiency in pediatric robot-assisted laparoscopic ureteric reimplantation: a learning curve configuration using cumulative sum analysis
Source: World J Urol. 2025 Jun 14;43(1):372. doi: 10.1007/s00345-025-05658-6 (PMC12167274; doi:10.1007/s00345-025-05658-6)
Supplement: Supplementary file 3 — Supplementary file3 (PDF 82 KB) [file 345_2025_5658_MOESM3_ESM.pdf]

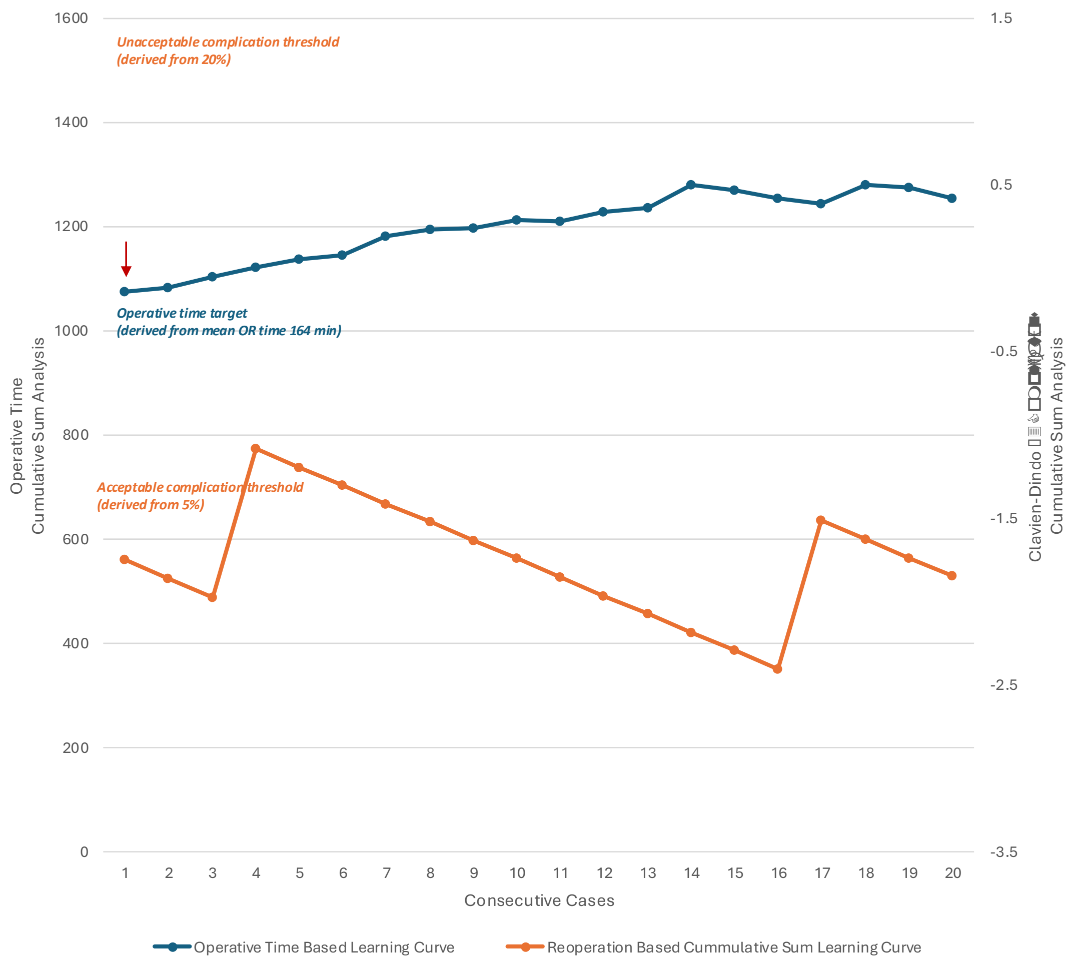

Supplementary Figure 3. Close-up detailed view of proficiency phase of the learning curve; complications are consistently below the acceptable threshold; operative time initially uptrends due to change in surgical technique at case indicated by red arrow (running to interrupted suturing) and plateaus as this technique is adopted
